# Association between rest-activity rhythm and cognitive function in the elderly: The U.S. National Health and Nutrition Examination Survey, 2011-2014

**DOI:** 10.3389/fendo.2023.1135085

**Published:** 2023-03-09

**Authors:** Xinyi Sun, Weiwei Yu, Mingsi Wang, Jun Hu, Yunong Li

**Affiliations:** ^1^ Department of Neurology, Peking University Shenzhen Hospital, Shenzhen, China; ^2^ National Key Discipline, Department of Nutrition and Food Hygiene, School of Public Health, Harbin Medical University, Harbin, China; ^3^ Department of Health Economics, College of Health Management of Harbin Medical University, Harbin, China

**Keywords:** rest-activity rhythm, circadian rhythm, cognitive function, older adult, NHANES

## Abstract

**Background:**

Circadian rhythm plays an essential role in various physiological and pathological processes related to cognitive function. The rest-activity rhythm (RAR) is one of the most prominent outputs of the circadian system. However, little is known about the relationships between RAR and different domains of cognitive function in older adults. The purpose of this study was to examine the relationships between RAR and various fields of cognitive function in older Americans.

**Methods:**

This study included a total of 2090 older adults ≥ 60 years old from the National Health and Nutrition Examination Survey (NHANES) in 2011-2014. RAR parameters were derived from accelerometer recordings. Cognitive function was assessed using the word learning subtest developed by the Consortium to Establish a Registry for Alzheimer’s disease (CERAD W-L), the Animal Fluency Test (AFT) and the Digital Symbol Substitution Test (DSST). Linear regression was used to determine the relationships between RAR parameters (IS, IV, RA, L5, M10) and cognitive function scores (CERAD W-L, AFT, DSST).

**Results:**

After adjusting for potential confounders, lower IS and M10 were associated with lower CERAD W-L scores (*P*=0.033 and *P*=0.002, respectively). Weaker RA and higher L5 were associated with lower AFT scores (*P*<0.001 and *P*=0.001, respectively). And lower IS, RA, and higher L5 were associated with lower DSST scores (*P*=0.019, *P*<0.001 and *P*<0.001, respectively). In addition, the results of sensitivity analysis were similar to those of our main analyses. The main correlation results between the RAR indicators and cognitive function were robust.

**Conclusions:**

This study suggested that the weakened and/or disrupted RAR was associated with cognitive decline in different domains in Americans over the age of 60.

## 1 Introduction

With the aging of the world population, the prevalence of cognitive impairment continues to rise. Various forms of dementia, from mild cognitive impairment (MCI) to Alzheimer’s disease (AD), are characterized by cognitive impairment and are rapidly becoming a serious global public health problem (1). About 40 million people worldwide are suffering from AD, and the number is expected to increase to 50 million by 2030 (2). To date, there is still no effective treatment to reverse or slow down the progress of cognitive impairment (3).

A large number of studies have shown that different lifestyles may have an impact on the cognitive function of the elderly. The Rancho Bernardo study shows that older adults with regular physical activity have better cognitive function. Physical activity in adolescence may enhance cognitive reserve to prevent age-related decline in executive function (4). The European Prospective Investigation into Cancer and Nutrition Norfolk (EPIC Norfolk) study shows that higher MedDiet adherence is related to better cognitive function and lower risk of cognitive impairment, especially among the elderly with higher CVD risk (5). Unhealthy lifestyles like smoking and drinking can also lead to cognitive impairment. Both secondhand and active smokers seemed to perform worse on cognitive tests, work and memory than nonsmoking peers (6, 7). A cohort study in China shows that poor lifestyle choices, such as drinking, unbalanced diet, low activity participation and air pollution, impair cognitive function in older adults. It is recommended that the elderly should avoid drinking, maintain a balanced diet, exercise more and pay attention to the impact of air quality (8).

In addition, circadian rhythm is an essentially biological process regulated by circadian clock genes and plays an important role in various physiological and pathological processes related to cognitive function (9). A blunted circadian rhythm increases the speed of biological aging. Dysregulation or disruption of circadian rhythms can also lead to health problems, including hypertension, sleep disorders, diabetes and cardiovascular disease (10). In the laboratory environment, severe circadian dysregulation will interfere with immune homeostasis, which plays a crucial role in the inflammatory process (11). RAR is one of the most prominent outputs of the circadian rhythm system (12). Several measurements calculated from rest-activity cycles are considered to reflect mild long-term disruption of circadian rhythms in real-world settings and can be objectively evaluated by accelerometers. In a recent prospective study, the accelerometer derived RAR measures, including amplitude, acrophase and pseudo-F statistic independently predicted the increased risk of diabetes among older adults (13). Diabetes is associated with poor cognitive function (executive function and processing speed) (14). Relevant studies have shown that indicators of RAR irregularity, such as reduced interdaily stability (IS) and increased intradaily variability (IV), are linked with an increased risk of neurodegenerative diseases, emphasizing the critical role of RAR in human health (15).

However, little is known about the associations of RAR with different domains of cognitive function in the U.S. elderly population. Therefore, we aimed to investigate the relationships between the RARs generated by accelerometric measurements and different fields of cognitive function in a large sample of older Americans using the NHANES in 2011-2014.

## 2 Materials and methods

### 2.1 Study population

Data were obtained from NHANES, a major program conducted by the National Center for Health Statistics (NCHS) to collect information on U.S. household population health and nutrition. The project includes two parts: family interview and physical examination. Details on sampling methods, survey tools and data collection are described elsewhere. All participants provided informed consent prior to participation (16). Our sample includes the elderly ≥ 60 years old, who have verified accelerometer records for at least 4 days and cognitive function data in the NHANES 2011-2014 cycle datasets.

### 2.2 Baseline data collection

During each NHANES cycle, data were collected through physical examinations and participant interviews. Participant interviews collected self-reported data on age (continuous), race (Mexican American, other Hispanic, non-Hispanic white, non-Hispanic black and others), sex (male/female), current smoker (yes/no), current drinker (yes/no), education levels (less than 9th grade/9-11th grade/high school graduate or equivalent/some college or AA degree/college graduate or above), sleep duration (hours), regular exercises habitus (yes/no), daily energy intake (kcal/d), annual household income (<$100,000 or > $100,000), whether to take medication for hypertension (yes/no), whether to take medication for diabetes (yes/no) and whether to take medication for cholesterol (yes/no). Hypertension, diabetes and hyperlipidemia were defined as self-reported hypertension, diabetes and hyperlipidemia, respectively. The NHANES examination data contain height and weight measurements used to calculate body mass index (BMI). All information regarding these methods is publicly available on the NHANES website.

### 2.3 Measurement of RAR

The R package “nparACT” is used to calculate the following non-parametric variables of RAR, which have been widely described before (17, 18). Interdaily stability (IS; value range 0 to 1), the stability index of day-to-day rest-activity mode; the larger the value, the more stable and consistent RAR across days (IS ≃ 0 represents Gaussian noise, and IS ≃ 1 represents perfect stability). Intradaily variability (IV; value range: 0 to 2), which reflects the index of 24-hour RAR fragmentation. The higher the value, the more significant the RAR disruption (IV ≃ 0 represents perfect sine wave, and IV ≃ 2 represents Gaussian noise). The relative amplitude (RA) is the relative difference between the most active continuous 10-hour period (M10) and the least active continuous 5-hour period (L5) in the average 24 hours (midnight to midnight). The higher the RA is, the stronger the 24-hour rest-activity oscillation is, reflecting that the activity is higher when awake, and the activity is relatively lower at night. L5 was defined as the average number of minutes of activity during the least active 5 hours of sleep over 24 hours, and M10 was defined as the average number of minutes of activity during the most active 10 hours per 24 hours.

### 2.4 The assessment of cognitive function

In the NHANES study, cognitive function was assessed using the word learning and recall modules developed by the Consortium to Establish a Registry for Alzheimer’s disease (CERAD W-L), the Animal Fluency Test (AFT) and the Digital Symbol Substitution Test (DSST). Although the results of these tests cannot replace the diagnosis based on clinical examination, they have been used to study the relationships between cognitive function and various risk factors (19).

CERAD W-L consists of three consecutive learning experiments and one delayed recall (20). Participants were asked to read 10 unrelated words aloud in three learning experiments. After the words were presented, participants immediately recalled as many words as possible. The delayed recall occurred about 10 minutes after the beginning of the word-learning experiment. The maximum score of each test is 10, and the highest score for the total word list was 40 (the sum of the three trials plus the delayed recall).

AFT is a language fluency task that checks execution functions (21). Participants were asked to name as many animals as possible within one minute. Each correctly named animal gets the point. Before the completion of the primary test, an exercise test was conducted in NHANES, asking participants to name three clothes, and then the main test was completed.

DSST is a performance module of the Wechsler Adult Intelligence Scale III (WAIS-III), which requires the integrity of executive function, processing speed, attention, spatial perception and visual scanning (22). The test was performed using a paper form with a key at the top containing 9 numbers and different symbols. Participants had two minutes to copy the corresponding symbols in 133 boxes adjacent to the number. The total number of correct matches determines the score, ranging from 0 to 133. Higher scores indicate a better cognitive function in each scoring dimension of cognitive function.

### 2.5 Stratified analysis

Stratified analysis is a subgroup analysis that divides participants in a study into groups based on different levels of relevant covariates and analyzes the strength of the associations between exposure factors and outcomes within each stratum. The study conducted the stratified analysis by age (60-69, ≥70), sex (male, female), BMI (<25.0, 25.0-29.9, ≥30.0) and race (Mexican American, other Hispanic, non-Hispanic white, non-Hispanic black and others).

### 2.6 Sensitivity analysis

We also performed several sensitivity analyses by excluding the elderly who had less than a 9th-grade education level; excluding those who reached peak activity levels between 23:00 and 04:00; and excluding those who had 6 hours or less sleep duration.

### 27 Statistical analysis

Continuous variables are expressed as mean ± standard deviation, and categorical variables are expressed as numbers with percentages. Associations of RAR with cognitive function were assessed by general linear regression analysis. We built three models to provide statistical inference. Model 1 was only adjusted for sex (male or female), age (continuous) and race (Mexican American, other Hispanic, non-Hispanic white, non-Hispanic black and others). Model 2 was adjusted for variables in model 1 plus BMI (kg/m^2^), income, education levels, sleep duration, daily energy intake, regular exercises, current smoker and current drinker. Model 3 was adjusted for variables in model 2 plus self-reported diabetes, self-reported hypertension, self-reported hyperlipidemia, take medication for diabetes, take medication for hypertension and take medication for cholesterol.

For all analyses, *P*<0.05 was considered statistically significant. All data analyses were performed by R Language (version 4.2.1).

## 3 Result

### 3.1 Characteristics of the study population

The general characteristic information of participants and RAR indicators are presented in **Table 1**. Our analysis included a total of 2090 older participants ≥ 60 years old (mean ± SD: 69.0 ± 6.7). Of the total participants, 47.4% were male, and 47.1% were non-Hispanic white, with a mean BMI of 29.11 ± 6.24 kg/m^2^; 14.2% of participants had an annual household income more than $100,000, and 23.5% had a college degree or above education. Other information, such as lifestyle, disease status, drug intervention information, RAR parameters and cognitive scores, are also summarized in **Table 1**.

**Table 1:** Descriptive characteristics of the study sample, NHANES 2011-2014.

Characteristics	All participants (N=2090)
Age, years, mean (SD)	69.0(6.7)
Male, n (%)	990(47.4%)
Non-Hispanic White, n (%)	984(47.1%)
BMI, kg/m^2^, mean (SD)	29.11(6.24)
>$100,000 annual household income, n (%)	297(14.2%)
College graduate or above, n (%)	492(23.5%)
Sleep duration, hours, mean (SD)	7.01(1.39)
Daily energy intake, kcal/day, mean (SD)	1822.44(670.24)
Exercise regularly, n (%)	1168(55.9%)
Current smoker, n (%)	272(13.0%)
Current drinker, n (%)	1431(68.5%)
Self-reported diabetes, n (%)	441(21.1%)
Self-reported hypertension, n (%)	1249(59.8%)
Self-reported hyperlipidemia, n (%)	1134(54.3%)
Take medication for diabetes, n (%)	108(5.2%)
Take medication for hypertension, n (%)	1094(52.3%)
Take medication for cholesterol, n (%)	812(38.9%)
Rest-activity parameters, mean (SD)	
IS	0.52(0.13)
IV	0.68(0.22)
RA	0.83(0.12)
L5	1.09(0.84)
M10	11.80(3.70)
CERAD W-L, mean (SD)	25.36(6.48)
AFT, mean (SD)	16.99(5.41)
DSST, mean (SD)	47.45(17.25)

Continuous variables are presented as mean (SD, standard deviation). Categorical variables are presented as numbers (%, percentage).

### 3.2 Association of RAR with cognitive function

The associations of RAR parameters with cognitive assessment scores are presented in **Table 2**. In regression models adjusted for covariates, the more stable RAR was significantly associated with better cognitive function. For the CERAD W-L scores, lower IS and M10 are significantly associated with lower CERAD W-L scores (IS: β=2.221, 95%CI: 0.175 to 4.246, *P*=0.033; M10: β=0.121, 95%CI: 0.044 to 0.199, *P*=0.002). For the AFT scores, weaker RA level is significantly correlated with lower AFT scores (β=3.406, 95%CI: 1.434 to 5.378, *P*<0.001), while higher L5 level is significantly associated with the decrease of the score (β=-0.448, 95%CI: -0.723 to -0.174, *P*=0.001). For the DSST scores, lower IS and RA levels are significantly correlated with lower DSST scores (IS: β=6.154, 95%CI: 0.999 to 11.308, *P*=0.019; RA: β= 20.449, 95%CI: 14.676 to 26.222, *P*<0.001), while higher L5 level is also significantly correlated with the decrease of the score (β=-2.361, 95%CI: -3.167 to -1.554, *P*<0.001). The analysis results showed no significant correlation between IV level and cognitive function scores (CERAD W-L, AFT and DSST). A stable RAR is associated with better cognitive performance, whereas disordered RAR may be associated with cognitive impairment.

**Table 2:** Associations of RAR parameters with cognitive function among all adults age≥60 years, NHANES 2011–2014.

		Unadjusted			Model 1			Model 2			Model 3		
		β	95%CI	P-value	β	95%CI	P-value	β	95%CI	P-value	β	95%CI	P-value
**CERAD W-L**	**IS**	1.591	(-0.537,3.719)	0.143	2.717	(0.688,4.746)	0.009	2.136	(0.100,4.172)	0.040	2.211	(0.175,4.246)	0.033
	**IV**	-2.495	(-3.783,-1.207)	<0.001	-0.626	(-1.884,0.631)	0.329	-0.458	(-1.719,0.804)	0.477	-0.407	(-1.668,0.855)	0.527
	**RA**	3.939	(1.568,6.309)	0.001	2.916	(0.646,5.186)	0.012	2.172	(-0.141,4.485)	0.066	2.195	(-0.121,4.512)	0.063
	**L5**	0.039	(-0.299,0.377)	0.821	-0.156	(-0.477,0.166)	0.343	-0.090	(-0.414,0.233)	0.584	-0.108	(-0.432,0.215)	0.512
	**M10**	0.311	(0.237,0.385)	<0.001	0.148	(0.073,0.224)	<0.001	0.127	(0.050,0.205)	0.001	0.121	(0.044,0.199)	0.002
**AFT**	**IS**	1.160	(-0.607,2.926)	0.198	1.314	(-0.440,3.069)	0.142	0.252	(-1.498,2.001)	0.778	0.291	(-1.457,2.038)	0.744
	**IV**	-1.705	(-2.775,-0.635)	0.002	-0.418	(-1.503,0.668)	0.450	-0.061	(-1.144,1.021)	0.911	-0.038	(-1.120,1.043)	0.944
	**RA**	5.544	(3.595,7.492)	<0.001	4.644	(2.700,6.588)	<0.001	3.625	(1.655,5.595)	<0.001	3.406	(1.434,5.378)	<0.001
	**L5**	-0.510	(-0.787,-0.233)	<0.001	-0.552	(-0.827,-0.277)	<0.001	-0.469	(-0.743,-0.195)	<0.001	-0.448	(-0.723,-0.174)	0.001
	**M10**	0.142	(0.079,0.204)	<0.001	0.056	(-0.009,0.121)	0.092	0.010	(-0.057,0.077)	0.767	0.006	(-0.060,0.073)	0.855
**DSST**	**IS**	7.304	(1.682,12.926)	0.011	11.187	(5.819,16.555)	<0.001	6.227	(1.049,11.405)	0.018	6.154	(0.999,11.308)	0.019
	**IV**	-5.985	(-9.392,-2.578)	<0.001	-1.193	(-4.525,2.140)	0.483	0.717	(-2.490,3.925)	0.661	1.060	(-2.134,4.254)	0.515
	**RA**	27.459	(21.315,33.602)	<0.001	26.489	(20.598,32.381)	<0.001	21.279	(15.494,27.063)	<0.001	20.449	(14.676,26.222)	<0.001
	**L5**	-2.208	(-3.089,-1.327)	<0.001	-2.899	(-3.737,-2.061)	<0.001	-2.438	(-3.247,-1.630)	<0.001	-2.361	(-3.167,-1.554)	<0.001
	**M10**	0.660	(0.462,0.858)	<0.001	0.234	(0.034,0.435)	0.022	0.003	(-0.195,0.201)	0.974	-0.014	(-0.211,0.183)	0.892

Model 1: adjusted for age, sex and race.

Model 2: adjusted for age, sex, race, BMI, income, education levels, sleep duration, daily energy intake, regular exercises, current smoker, and current drinker.

Model 3: adjusted for age, sex, race, BMI, daily energy intake, education levels, sleep duration, regular exercises, current smoker, current drinker, income, self-reported diabetes, self-reported hypertension, self-reported hyperlipidemia, take medication for diabetes, take medication for hypertension and take medication for cholesterol.

### 3.3 Stratified analysis

**Figure 1:**
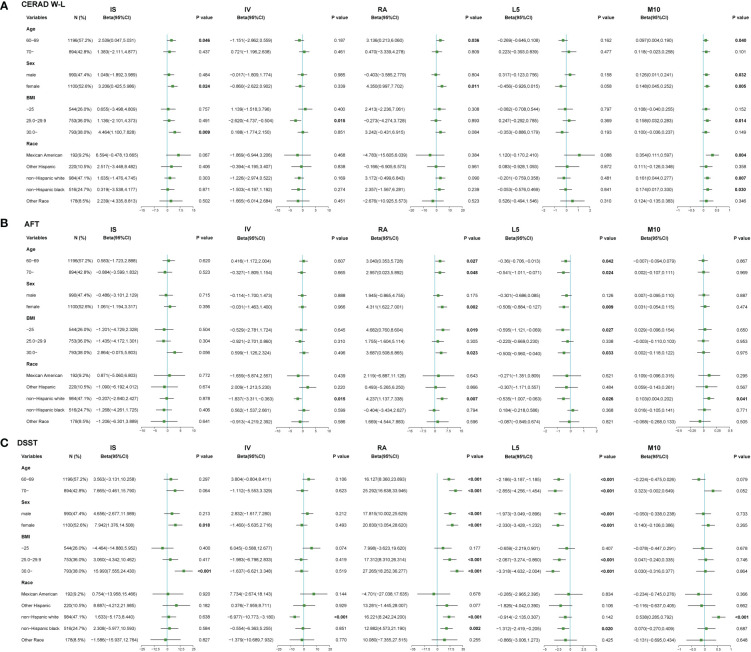
Relationships between RAR and cognitive function stratified by risk factors. **(A)** Relationships between RAR and CERAD W-L scores in stratified analysis. **(B)** Relationships between RAR and AFT scores in stratified analysis. **(C)** Relationships between RAR and DSST scores in stratified analysis. Results were adjusted for age (60-69, ≥70), sex (male, female), BMI (<25, 25-29.9, ≥30 kg/m^2^), race (Mexican American, other Hispanic, non-Hispanic white, non-Hispanic black, others). Dots indicate beta estimates, and black horizontal lines indicate 95% CIs.

We performed a stratified subgroup analysis of the relationships between RARs and cognitive function scores by age, sex, BMI and race. The results of stratified analysis were shown in **Figure 1** and **Supplementary Tables 1**-**3**.

### 3.4 Sensitivity analysis

In the first two sensitivity analyses, after excluding participants who had less than a 9th-grade education level or those who reached peak activity levels between 23:00 and 04:00, the results of the relationships between RAR and cognitive function were basically the same as those of the main analysis, except that the significant correlations between IS and CERAD W-L scores, and the significant correlations between IS and DSST scores disappeared (**Supplementary Tables 4**, **5**). In the third sensitivity analysis, after excluding participants with 6 hours or less sleep duration, the correlations between RAR and cognitive function remained similar to the main results, except that the correlations between IS and CERAD W-L scores disappeared (**Supplementary Table 6**). We observed that, except for the unstable correlations of IS on cognitive function, the main correlation results of other RAR indicators on cognitive function were robust. Although the results of sensitivity analyses show that the exclusion of some factor variables has no significant impact on our results, it may reduce the universal representativeness of our research samples.

## 4 Discussion

For the first time, we used the sample of older American adults to test whether RAR disturbance measured from accelerometer data is associated with cognitive function. We found that impaired circadian rhythm (IS, RA), lower M10 values, and higher L5 values were associated with cognitive decline in different domains. Irregular rest-activity patterns were associated with poor memory (CERAD W-L), executive function (AFT), processing speed and global cognitive function (DSST). In order to avoid the false correlation caused by confounding factors, we further controlled the confounding factors, and the research results remained unchanged; that is, impaired robustness of RAR was related to the decline of cognitive function. In different models, we did not observe significant results in the association of fragmented RAR (estimated by IV) with cognitive decline, suggesting that our bodies may have higher levels of adaptive ability in the rhythm phase shift on daily schedules. These findings suggest that RAR disturbance may be a sign of impaired memory formation and cognitive slowing, which can predict the cognitive function of the elderly, and is also a potential therapeutic target.

In this study, we examined the relationships between different parameters of RAR and cognitive function. RA assesses the overall robustness of the RAR, with larger RA indicating that individuals are more active during the day and less active at night. It has been found to be a sensitive indicator of RAR dysregulation. This parameter reflects the long-term comprehensive influence of environmental and behavioral factors on the circadian rhythm system. Several studies have shown that RA is associated with dementia-related biomarkers such as T-tau and NF-L (23–25). The levels of these biomarkers were reduced in patients with higher amplitudes, implying that higher RA was negatively correlated with cognitive decline, which is consistent with the findings of this study. In addition, previous studies have observed that lower RA is closely related to diseases such as abnormal glucose tolerance and diabetes (13, 26, 27). Compared with normoglycemic older adults, the elderly with diabetes were more likely to have MCI. This also confirmed our results.

Our study found that a weaker daytime stability (lower IS) was associated with decreased cognitive function. Van Someren et al. suggested maintaining a regular rhythm, adhering to a stricter 24-hour clock and enhancing daytime stability could protect the fragile circadian system in the elderly population and reduce the risk of cognitive impairment (28). We also observed that lower M10 values were associated with poor cognitive function (CERAD W-L), and higher L5 was associated with more inferior cognitive function (AFT, DSST). Several studies have shown that lower M10 values representing lower daily activity indices and higher L5 values representing higher nocturnal activity indices are associated with increased inflammatory markers (29–31). A recent pre-clinical study reported that neuroinflammation exacerbates cognitive impairment in a rat model of vascular dementia (32).

In this study, we also examined the relationships between RAR and different cognitive domains. DSST is considered as a sensitive indicator of global cognitive function, which depends on processing speed, visual scanning, sustained attention and short-term memory. Compared with older adults with normal RAR, older adults with RAR disorder had lower DSST scores. Previous studies have shown that the negative impact of sleep or circadian rhythm disruption on processing speed has been shown in shift workers and older adults in the general population (33). The results from the CERAD W-L measurement (word learning and short-term memory test) show that RAR disturbance may affect memory function, which means that memory structures in the brain, including hippocampus, may be damaged (34). Smagula et al. showed that RAR disruption is associated with hippocampal hyperactivation, which was considered to be one of the pathophysiological changes of early memory decline related to pre-clinical dementia (35). In animal experiments and epidemiological studies, it has been reported that work and memory function decreased due to insufficient sleep or circadian rhythm disorder (36, 37). Furthermore, in addition to CERAD and DSST, we included the AFT score representing executive function as an outcome variable in the statistical model, and the results showed that impaired RAR was associated with decreased executive function. RAR disorders may affect different areas of cognitive function by affecting multiple brain functional areas.

Several studies have investigated whether mild intervention with RAR could change the process of cognitive decline and protect brain health. Interventions included timed BLT, consistent sleep-wake times (avoiding shift work, jet lag and naps), behavioral rhythm interventions at mealtime and other activity schedules, increasing or maintaining activity participation, avoiding low daytime light and night light and appropriately timed bright daytime light. These behavioral interventions are low risk, easy to spread, and have no drug interactions or side effects. These interventions may help to strengthen the circadian rhythm to improve rhythm stability and amplitude as well as cognitive function. For example, bright light therapy can reduce the cognitive decline in elderly residents over the course of one year (38), and 4-week doses of bright light therapy could improve MMSE scores in AD dementia patients (39). However, some studies also showed that bright light changed the RAR in dementia patients, but not the course of dementia. Interventions targeting RAR may need to target specific subgroups or use multiple approaches (40, 41).

This study shows several strengths. First, this study used the sample of older adults from NHANES with high-quality survey methods and quality control. Most of the participants had high compliance with equipment wearing and long equipment wearing time. Second, the cognitive data includes three different cognitive tests, providing more and deeper information than the previous NHANES surveys. In addition, we controlled for multiple important confounders to estimate the correlations between RAR and cognitive function.

Nonetheless, there are some limitations to our study. First, cross-sectional design may lead to a lack of causal relationships between RAR and cognitive function. Second, although we adjusted for a wide range of confounders, unmeasured biomarkers may lead to residual confounders. In addition, the shift work status data was not obtained from the participants in the NHANES 2011-2014, so the sensitivity test to exclude these participants could not be carried out. However, excluding those with peak activity at night (i.e., between 23:00 and 04:00) did not change our results, suggesting that the potential impact of shift work on our study would be minimal. The results still need to be confirmed in the future longitudinal study of randomized intervention.

## 5 Conclusion

This study investigated the relationships between rest-activity rhythm and cognitive function, demonstrating the importance of circadian rhythm on cognitive function in the elderly population. After adjusting for a range of confounders, we found that lower levels of IS, RA and M10, and higher levels of L5 were significantly associated with lower cognitive scores across different domains. Our results provide a new basis for future efforts to improve cognition in older adults by changing daily personal behaviors and lifestyles to influence circadian rhythm.

## Data availability statement

The original contributions presented in the study are included in the article/**Supplementary Material**. Further inquiries can be directed to the corresponding authors.

## Ethics statement

The studies involving human participants were reviewed and approved by the study protocol approved by the NCHS Research Ethics Review Board. The patients/participants provided their written informed consent to participate in this study.

## Author contributions

YL, JH, and MW designed the analysis. WY and XS wrote the first draft of the article. XS conducted the statistical analyses. YL and MW revised the manuscript. All authors contributed to the article and approved the submitted version.
