# Supplementary material for: Association between rest-activity rhythm and cognitive function in the elderly: The U.S. National Health and Nutrition Examination Survey, 2011-2014
Source: Front Endocrinol (Lausanne). 2023 Mar 9;14:1135085. doi: 10.3389/fendo.2023.1135085 (PMC10034093; doi:10.3389/fendo.2023.1135085)
Supplement: Supplementary file 1 [file Table_1.docx]

***Supplementary Material***

**Association between rest-activity rhythm and cognitive function in the elderly: the U.S. National Health and Nutrition Examination Survey, 2011-2014**

**Xinyi Sun^1，2#^, Weiwei Yu^1#^, Mingsi Wang^3*^, Jun Hu^1*^ and Yunong Li^1*^**

^1^ Department of Neurology, Peking University Shenzhen Hospital, Shenzhen, China, 518036

^2^ National Key Discipline, Department of Nutrition and Food Hygiene, School of Public Health, Harbin Medical University, Harbin, China 150081

^3^ Department of Health Economics, College of Health Management of Harbin Medical University, Harbin, China, 150086.

*** Correspondence:**

1. Yunong Li: Department of Neurology, Peking University Shenzhen Hospital, Shenzhen, China, 518036. E-mail: [yunong_li@hrbmu.edu.cn](mailto:yunong_li@hrbmu.edu.cn).

2. Jun Hu: Department of Neurology, Peking University Shenzhen Hospital, Shenzhen, China, 518036. E-mail: dochj@163.com.

3. Mingsi Wang: Department of Health Economics, College of Health Management of Harbin Medical University, Harbin, China, 150086. E-mail: [wangmingsi@hrbmu.edu.cn](mailto:wangmingsi@hrbmu.edu.cn)

# Xinyi Sun and Weiwei Yu are co-first authors who equally contributed to this work.

**Keywords: rest-activity rhythm, circadian rhythm, cognitive function, older adult,** **NHANES.**

**Supplementary Table 1:** **Associations of RAR parameters with the CERAD W-L scores in stratified subgroup analysis.**

|  |  |  | IS-CERAD | | IV-CERAD | | RA-CERAD | | L5-CERAD | | M10-CERAD | |
| --- | --- | --- | --- | --- | --- | --- | --- | --- | --- | --- | --- | --- |
|  | Characteristics | N (%) | β (95% CI) | P | β (95% CI) | P | β (95% CI) | P | β (95% CI) | P | β (95% CI) | P |
| Age | 60-69 | 1196(57.2%) | 2.539(0.047,5.031) | 0.046 | -1.151(-2.862,0.559) | 0.187 | 3.136(0.213,6.060) | 0.036 | -0.269(-0.646,0.108) | 0.162 | 0.097(0.004,0.190) | 0.040 |
|  | ≥70 | 894(42.8%) | 1.383(-2.111,4.877) | 0.437 | 0.721(-1.196,2.638) | 0.461 | 0.470(-3.339,4.278) | 0.809 | 0.223(-0.393,0.839) | 0.477 | 0.118(-0.023,0.258) | 0.101 |
| Sex | male | 990(47.4%) | 1.049(-1.892,3.989) | 0.484 | -0.017(-1.809,1.774) | 0.985 | -0.403(-3.585,2.779) | 0.804 | 0.317(-0.123,0.756) | 0.158 | 0.126(0.011,0.241) | 0.032 |
|  | female | 1100(52.6%) | 3.206(0.425,5.986) | 0.024 | -0.860(-2.622,0.902) | 0.339 | 4.350(0.997,7.702) | 0.011 | -0.456(-0.926,0.015) | 0.058 | 0.148(0.045,0.252) | 0.005 |
| BMI | <25.0 | 544(26.0%) | 0.655(-3.498,4.809) | 0.757 | 1.139(-1.518,3.796) | 0.400 | 2.413(-2.236,7.061) | 0.308 | -0.082(-0.708,0.544) | 0.797 | 0.108(-0.040,0.255) | 0.152 |
|  | 25.0-29.9 | 753(36.0%) | 1.136(-2.101,4.373) | 0.491 | -2.620(-4.737,-0.504) | 0.015 | -0.273(-4.274,3.728) | 0.893 | 0.247(-0.292,0.785) | 0.369 | 0.158(0.032,0.283) | 0.014 |
|  | ≥30.0 | 793(38.0%) | 4.464(1.100,7.828) | 0.009 | 0.188(-1.774,2.150) | 0.851 | 3.242(-0.431,6.915) | 0.084 | -0.353(-0.886,0.179) | 0.193 | 0.100(-0.036,0.237) | 0.149 |
| Race | Mexican American | 192(9.2%) | 6.594(-0.478,13.665) | 0.067 | -1.869(-6.944,3.206) | 0.468 | -4.783(-15.605,6.039) | 0.384 | 1.120(-0.170,2.410) | 0.088 | 0.354(0.111,0.597) | 0.004 |
|  | Other Hispanic | 220(10.5%) | 2.517(-3.448,8.482) | 0.406 | -0.394(-4.195,3.407) | 0.838 | -0.166(-6.905,6.573) | 0.961 | 0.083(-0.928,1.093) | 0.872 | 0.111(-0.126,0.348) | 0.358 |
|  | non-Hispanic white | 984(47.1%) | 1.635(-1.476,4.745) | 0.303 | -1.226(-2.974,0.522) | 0.169 | 3.172(-0.499,6.843) | 0.090 | -0.201(-0.759,0.358) | 0.481 | 0.161(0.044,0.277) | 0.007 |
|  | non-Hispanic black | 516(24.7%) | 0.319(-3.538,4.177) | 0.871 | -1.503(-4.197,1.192) | 0.274 | 2.357(-1.567,6.281) | 0.239 | -0.053(-0.576,0.469) | 0.841 | 0.174(0.017,0.330) | 0.030 |
|  | Other Race | 178(8.5%) | 2.239(-4.335,8.813) | 0.502 | -1.665(-6.014,2.684) | 0.451 | -2.676(-10.925,5.573) | 0.523 | 0.526(-0.494,1.546) | 0.310 | 0.124(-0.135,0.383) | 0.346 |

Age subgroups are adjusted for sex, race, BMI, daily energy intake, education levels, sleep duration, regular exercises, current smoker, current drinker, income, self-reported diabetes, self-reported hypertension, self-reported hyperlipidemia, take medication for diabetes, take medication for hypertension and take medication for cholesterol.

Sex subgroups are adjusted for age, race, BMI, daily energy intake, education levels, sleep duration, regular exercises, current smoker, current drinker, income, self-reported diabetes, self-reported hypertension, self-reported hyperlipidemia, take medication for diabetes, take medication for hypertension and take medication for cholesterol.

BMI subgroups are adjusted for age, sex, race, daily energy intake, education levels, sleep duration, regular exercises, current smoker, current drinker, income, self-reported diabetes, self-reported hypertension, self-reported hyperlipidemia, take medication for diabetes, take medication for hypertension and take medication for cholesterol.

Race subgroups are adjusted for age, sex, BMI, daily energy intake, education levels, sleep duration, regular exercises, current smoker, current drinker, income, self-reported diabetes, self-reported hypertension, self-reported hyperlipidemia, take medication for diabetes, take medication for hypertension and take medication for cholesterol.

**Supplementary Table 2: Associations of RAR parameters with the AFT scores in stratified subgroup analysis.**

|  |  |  | IS-AFT | | IV-AFT | | RA-AFT | | L5-AFT | | M10-AFT | |
| --- | --- | --- | --- | --- | --- | --- | --- | --- | --- | --- | --- | --- |
|  | Characteristics | N (%) | β (95% CI) | P | β (95% CI) | P | β (95% CI) | P | β (95% CI) | P | β (95% CI) | P |
| Age | 60-69 | 1196(57.2%) | 0.583(-1.723,2.888) | 0.620 | 0.416(-1.172,2.004) | 0.607 | 3.040(0.353,5.728) | 0.027 | -0.360(-0.706,-0.013) | 0.042 | -0.007(-0.094,0.079) | 0.867 |
|  | ≥70 | 894(42.8%) | -0.884(-3.599,1.832) | 0.523 | -0.327(-1.809,1.154) | 0.665 | 2.957(0.023,5.892) | 0.048 | -0.541(-1.011,-0.071) | 0.024 | 0.002(-0.107,0.111) | 0.969 |
| Sex | male | 990(47.4%) | -0.486(-3.101,2.129) | 0.715 | -0.114(-1.700,1.473) | 0.888 | 1.945(-0.865,4.755) | 0.175 | -0.301(-0.686,0.085) | 0.126 | 0.007(-0.095,0.110) | 0.887 |
|  | female | 1100(52.6%) | 1.061(-1.194,3.317) | 0.356 | -0.031(-1.463,1.400) | 0.966 | 4.311(1.622,7.001) | 0.002 | -0.506(-0.884,-0.127) | 0.009 | 0.031(-0.054,0.115) | 0.474 |
| BMI | <25.0 | 544(26.0%) | -1.201(-4.729,2.328) | 0.504 | -0.529(-2.781,1.724) | 0.645 | 4.682(0.760,8.604) | 0.019 | -0.595(-1.121,-0.069) | 0.027 | 0.029(-0.096,0.154) | 0.650 |
|  | 25.0-29.9 | 753(36.0%) | -1.435(-4.172,1.301) | 0.304 | -0.921(-2.701,0.860) | 0.310 | 1.755(-1.604,5.114) | 0.305 | -0.220(-0.669,0.230) | 0.338 | -0.003(-0.110,0.103) | 0.953 |
|  | ≥30.0 | 793(38.0%) | 2.864(-0.075,5.803) | 0.056 | 0.599(-1.126,2.324) | 0.496 | 3.687(0.508,6.865) | 0.023 | -0.500(-0.960,-0.040) | 0.033 | 0.002(-0.118,0.122) | 0.975 |
| Race | Mexican American | 192(9.2%) | 0.871(-5.060,6.803) | 0.772 | -1.659(-5.874,2.557) | 0.439 | 2.119(-6.887,11.126) | 0.643 | -0.271(-1.351,0.809) | 0.621 | 0.109(-0.096,0.315) | 0.295 |
|  | Other Hispanic | 220(10.5%) | -1.090(-6.192,4.012) | 0.674 | 2.009(-1.213,5.230) | 0.220 | 0.493(-5.265,6.250) | 0.866 | -0.307(-1.171,0.557) | 0.484 | 0.059(-0.143,0.261) | 0.567 |
|  | non-Hispanic white | 984(47.1%) | -0.207(-2.840,2.427) | 0.878 | -1.837(-3.311,-0.363) | 0.015 | 4.237(1.137,7.338) | 0.007 | -0.535(-1.007,-0.063) | 0.026 | 0.103(0.004,0.202) | 0.041 |
|  | non-Hispanic black | 516(24.7%) | -1.268(-4.261,1.725) | 0.406 | 0.562(-1.537,2.661) | 0.599 | -0.404(-3.434,2.627) | 0.794 | 0.184(-0.218,0.586) | 0.368 | 0.018(-0.105,0.141) | 0.771 |
|  | Other Race | 178(8.5%) | -1.206(-6.301,3.889) | 0.641 | -0.913(-4.219,2.392) | 0.586 | 1.669(-4.544,7.883) | 0.596 | -0.087(-0.849,0.674) | 0.821 | -0.068(-0.268,0.133) | 0.505 |

Age subgroups are adjusted for sex, race, BMI, daily energy intake, education levels, sleep duration, regular exercises, current smoker, current drinker, income, self-reported diabetes, self-reported hypertension, self-reported hyperlipidemia, take medication for diabetes, take medication for hypertension and take medication for cholesterol.

Sex subgroups are adjusted for age, race, BMI, daily energy intake, education levels, sleep duration, regular exercises, current smoker, current drinker, income, self-reported diabetes, self-reported hypertension, self-reported hyperlipidemia, take medication for diabetes, take medication for hypertension and take medication for cholesterol.

BMI subgroups are adjusted for age, sex, race, daily energy intake, education levels, sleep duration, regular exercises, current smoker, current drinker, income, self-reported diabetes, self-reported hypertension, self-reported hyperlipidemia, take medication for diabetes, take medication for hypertension and take medication for cholesterol.

Race subgroups are adjusted for age, sex, BMI, daily energy intake, education levels, sleep duration, regular exercises, current smoker, current drinker, income, self-reported diabetes, self-reported hypertension, self-reported hyperlipidemia, take medication for diabetes, take medication for hypertension and take medication for cholesterol.

|  |  | |  | IS-DSST | | IV-DSST | | RA-DSST | | L5-DSST | | M10-DSST | |
| --- | --- | --- | --- | --- | --- | --- | --- | --- | --- | --- | --- | --- | --- |
|  | Characteristics | N (%) | | β (95% CI) | P | β (95% CI) | P | β (95% CI) | P | β (95% CI) | P | β (95% CI) | P |
| Age | 60-69 | 1196(57.2%) | | 3.563(-3.131,10.258) | 0.297 | 3.804(-0.804,8.411) | 0.106 | 16.127(8.360,23.893) | <0.001 | -2.186(-3.187,-1.185) | <0.001 | -0.224(-0.475,0.026) | 0.079 |
|  | ≥70 | 894(42.8%) | | 7.665(-0.461,15.790) | 0.064 | -1.112(-5.553,3.329) | 0.623 | 25.292(16.638,33.946) | <0.001 | -2.855(-4.256,-1.454) | <0.001 | 0.323(-0.002,0.649) | 0.052 |
| Sex | male | 990(47.4%) | | 4.656(-2.677,11.989) | 0.213 | 2.832(-1.617,7.280) | 0.212 | 17.815(10.002,25.629) | <0.001 | -1.973(-3.049, -0.896) | <0.001 | -0.050(-0.338,0.238) | 0.733 |
|  | female | 1100(52.6%) | | 7.942(1.376,14.508) | 0.018 | -1.460(-5.635,2.716) | 0.493 | 20.830(13.054,28.620) | <0.001 | -2.330(-3.428,-1.232) | <0.001 | 0.140(-0.106,0.386) | 0.265 |
| BMI | <25.0 | 544(26.0%) | | -4.464(-14.880,5.952) | 0.400 | 6.045(-0.588,12.677) | 0.074 | 7.998(-3.623,19.620) | 0.177 | -0.659(-2.219,0.901) | 0.407 | -0.078(-0.447,0.291) | 0.678 |
|  | 25.0-29.9 | 753(36.0%) | | 3.060(-4.342,10.462) | 0.417 | -1.983(-6.798,2.833) | 0.419 | 17.312(8.310,26.314) | <0.001 | -2.067(-3.274,-0.860) | <0.001 | 0.047(-0.240,0.335) | 0.746 |
|  | ≥30.0 | 793(38.0%) | | 15.993(7.555,24.430) | <0.001 | -1.637(-6.621,3.348) | 0.519 | 27.265(18.252,36.277) | <0.001 | -3.318(-4.632,-2.004) | <0.001 | 0.030(-0.316,0.377) | 0.864 |
| Race | Mexican American | 192(9.2%) | | 0.754(-13.958,15.466) | 0.920 | 7.734(-2.674,18.143) | 0.144 | -4.701(-27.038,17.635) | 0.678 | -0.285(-2.965,2.395) | 0.834 | -0.234(-0.745,0.276) | 0.366 |
|  | Other Hispanic | 220(10.5%) | | 8.887(-4.212,21.985) | 0.182 | 0.376(-7.959,8.711) | 0.929 | 13.281(-1.445,28.007) | 0.077 | -1.826(-4.042,0.390) | 0.106 | -0.116(-0.637,0.405) | 0.662 |
|  | non-Hispanic white | 984(47.1%) | | 1.633(-5.173,8.440) | 0.638 | -6.977(-10.773,-3.180) | <0.001 | 16.221(8.242,24.200) | <0.001 | -0.914(-2.135,0.307) | 0.142 | 0.538(0.285,0.792) | <0.001 |
|  | non-Hispanic black | 516(24.7%) | | 2.308(-5.977,10.593) | 0.584 | -0.554(-6.363,5.255) | 0.851 | 12.882(4.573,21.190) | 0.002 | -1.312(-2.419,-0.205) | 0.020 | 0.070(-0.270,0.409) | 0.687 |
|  | Other Race | 178(8.5%) | | -1.586(-15.937,12.764) | 0.827 | -1.379(-10.689,7.932) | 0.770 | 10.080(-7.355,27.515) | 0.255 | -0.866(-3.006,1.273) | 0.425 | -0.131(-0.695,0.434) | 0.648 |

**Supplementary Table 3: Associations of RAR parameters with the DSST scores in stratified subgroup analysis.**

Age subgroups are adjusted for sex, race, BMI, daily energy intake, education levels, sleep duration, regular exercises, current smoker, current drinker, income, self-reported diabetes, self-reported hypertension, self-reported hyperlipidemia, take medication for diabetes, take medication for hypertension and take medication for cholesterol.

Sex subgroups are adjusted for age, race, BMI, daily energy intake, education levels, sleep duration, regular exercises, current smoker, current drinker, income, self-reported diabetes, self-reported hypertension, self-reported hyperlipidemia, take medication for diabetes, take medication for hypertension and take medication for cholesterol.

BMI subgroups are adjusted for age, sex, race, daily energy intake, education levels, sleep duration, regular exercises, current smoker, current drinker, income, self-reported diabetes, self-reported hypertension, self-reported hyperlipidemia, take medication for diabetes, take medication for hypertension and take medication for cholesterol.

Race subgroups are adjusted for age, sex, BMI, daily energy intake, education levels, sleep duration, regular exercises, current smoker, current drinker, income, self-reported diabetes, self-reported hypertension, self-reported hyperlipidemia, take medication for diabetes, take medication for hypertension and take medication for cholesterol.

**Supplementary Table 4: Associations of RAR parameters with cognitive function excluding participants with** **less than a 9th-grade education level. (N=1875)**

|  |  | **Unadjusted** | | | **Model 1** | | | **Model 2** | | | **Model 3** | | |
| --- | --- | --- | --- | --- | --- | --- | --- | --- | --- | --- | --- | --- | --- |
|  |  | β | 95%CI | P-value | β | 95%CI | P-value | β | 95%CI | P-value | β | 95%CI | P-value |
| **CERAD W-L** | **IS** | 0.909 | (-1.306,3.124) | 0.421 | 2.093 | (-0.031,4.217) | 0.053 | 1.642 | (-0.510,3.794) | 0.135 | 1.692 | (-0.461,3.846) | 0.123 |
|  | **IV** | -3.440 | (-4.783,-2.097) | < <0.001 | -1.462 | (-2.775,-0.150) | 0.029 | -1.172 | (-2.501,0.158) | 0.084 | -1.106 | (-2.435,0.224) | 0.103 |
|  | **RA** | 3.963 | (1.540,6.387) | 0.001 | 2.749 | (0.423,5.075) | 0.021 | 2.213 | (-0.177,4.604) | 0.070 | 2.227 | (-0.167,4.620) | 0.068 |
|  | **L5** | 0.005 | (-0.336,0.347) | 0.976 | -0.131 | (-0.457,0.195) | 0.429 | -0.101 | (-0.432,0.230) | 0.550 | -0.121 | (-0.452,0.210) | 0.473 |
|  | **M10** | 0.335 | (0.257,0.414) | <0.001 | 0.171 | (0.092,0.250) | <0.001 | 0.147 | (0.065,0.229) | <0.001 | 0.138 | (0.056,0.221) | 0.001 |
| **AFT** | **IS** | 1.360 | (-0.527,3.247) | 0.158 | 1.335 | (-0.531,3.200) | 0.161 | 0.297 | (-1.576,2.169) | 0.756 | 0.316 | (-1.555,2.186) | 0.741 |
|  | **IV** | -2.460 | (-3.609,-1.311) | <0.001 | -1.185 | (-2.339,-0.031) | 0.044 | -0.703 | (-1.861,0.455) | 0.234 | -0.689 | (-1.845,0.467) | 0.243 |
|  | **RA** | 5.920 | (3.872,7.968) | <0.001 | 4.789 | (2.761,6.816) | <0.001 | 3.863 | (1.796,5.930) | <0.001 | 3.622 | (1.555,5.689) | <0.001 |
|  | **L5** | -0.528 | (-0.817,-0.240) | <0.001 | -0.531 | (-0.815,-0.246) | <0.001 | -0.470 | (-0.756,-0.185) | 0.001 | -0.449 | (-0.735,-0.164) | 0.002 |
|  | **M10** | 0.170 | (0.102,0.238) | <0.001 | 0.082 | (0.012,0.152) | 0.022 | 0.026 | (-0.046,0.098) | 0.479 | 0.021 | (-0.051,0.092) | 0.574 |
| **DSST** | **IS** | 6.558 | (1.000,12.116) | 0.021 | 9.391 | (4.093,14.688) | <0.001 | 5.152 | (-0.049,10.354) | 0.052 | 5.038 | (-0.151,10.227) | 0.057 |
|  | **IV** | -9.891 | (-13.264,-6.518) | <0.001 | -4.409 | (-7.692,-1.126) | 0.009 | -2.243 | (-5.463,0.976) | 0.172 | -2.081 | (-5.292,1.130) | 0.204 |
|  | **RA** | 28.454 | (22.502,34.405) | <0.001 | 25.356 | (19.665,31.048) | <0.001 | 21.355 | (15.669,27.041) | <0.001 | 20.576 | (14.895,26.258) | <0.001 |
|  | **L5** | -2.265 | (-3.113,-1.418) | <0.001 | -2.643 | (-3.446,-1.840) | <0.001 | -2.385 | (-3.175,-1.596) | <0.001 | -2.318 | (-3.106,-1.529) | <0.001 |
|  | **M10** | 0.838 | (0.641,1.035) | <0.001 | 0.378 | (0.180,0.577) | <0.001 | 0.127 | (-0.073,0.327) | 0.213 | 0.108 | (-0.091,0.308) | 0.287 |

Model 1: adjusted for age, sex and race.

Model 2: adjusted for age, sex, race, BMI, income, education levels, sleep duration, daily energy intake, regular exercises, current smoker, and current drinker.

Model 3: adjusted for age, sex, race, BMI, daily energy intake, education levels, sleep duration, regular exercises, current smoker, current drinker, income, self-reported diabetes, self-reported hypertension, self-reported hyperlipidemia, take medication for diabetes, take medication for hypertension and take medication for cholesterol.

**Supplementary Table 5: Associations of RAR parameters with cognitive function excluding participants who reached peak activity levels between 23:00 and 04:00. (N=1611)**

|  |  | **Unadjusted** | | | **Model 1** | | | **Model 2** | | | **Model 3** | | |
| --- | --- | --- | --- | --- | --- | --- | --- | --- | --- | --- | --- | --- | --- |
|  |  | β | 95%CI | P-value | β | 95%CI | P-value | β | 95%CI | P-value | β | 95%CI | P-value |
| **CERAD W-L** | **IS** | 0.947 | (-1.438,3.333) | 0.436 | 2.318 | (0.046,4.591) | 0.046 | 1.726 | (-0.556,4.008) | 0.138 | 1.817 | (-0.463,4.098) | 0.118 |
|  | **IV** | -2.718 | (-4.183,-1.254) | < <0.001 | -1.031 | (-2.456,0.395) | 0.156 | -0.980 | (-2.403,0.444) | 0.177 | -0.952 | (-2.376,0.471) | 0.189 |
|  | **RA** | 4.084 | (1.426,6.743) | 0.003 | 3.192 | (0.640,5.744) | 0.014 | 2.428 | (-0.174,5.029) | 0.067 | 2.469 | (-0.135,5.074) | 0.063 |
|  | **L5** | -0.045 | (-0.416,0.327) | 0.814 | -0.229 | (-0.584,0.125) | 0.205 | -0.150 | (-0.507,0.206) | 0.408 | -0.170 | (-0.527,0.187) | 0.350 |
|  | **M10** | 0.296 | (0.210,0.381) | <0.001 | 0.144 | (0.058,0.230) | 0.001 | 0.119 | (0.031,0.206) | 0.008 | 0.113 | (0.025,0.200) | 0.012 |
| **AFT** | **IS** | 0.442 | (-1.568,2.452) | 0.667 | 0.773 | (-1.209,2.755) | 0.444 | -0.424 | (-2.397,1.549) | 0.673 | -0.413 | (-2.385,1.559) | 0.681 |
|  | **IV** | -1.658 | (-2.894,-0.422) | 0.009 | -0.266 | (-1.509,0.977) | 0.674 | 0.026 | (-1.205,1.257) | 0.967 | 0.060 | (-1.171,1.290) | 0.924 |
|  | **RA** | 5.620 | (3.406,7.834) | <0.001 | 4.450 | (2.247,6.653) | <0.001 | 3.339 | (1.113,5.566) | 0.003 | 3.126 | (0.896,5.356) | 0.006 |
|  | **L5** | -0.505 | (-0.813,-0.196) | 0.001 | -0.514 | (-0.819,-0.209) | <0.001 | -0.401 | (-0.705,-0.096) | 0.010 | -0.379 | (-0.684,-0.075) | 0.015 |
|  | **M10** | 0.133 | (0.060,0.205) | <0.001 | 0.037 | (-0.038,0.112) | 0.332 | -0.009 | (-0.085,0.066) | 0.808 | -0.014 | (-0.089,0.062) | 0.726 |
| **DSST** | **IS** | 4.678 | (-1.682,11.038) | 0.149 | 8.871 | (2.797,14.945) | 0.004 | 3.426 | (-2.385,9.237) | 0.248 | 3.288 | (-2.500,9.075) | 0.265 |
|  | **IV** | -3.820 | (-7.738,0.098) | 0.056 | 0.619 | (-3.200,4.438) | 0.751 | 2.166 | (-1.460,5.792) | 0.241 | 2.461 | (-1.151,6.072) | 0.182 |
|  | **RA** | 26.154 | (19.207,33.102) | <0.001 | 25.375 | (18.691,32.059) | <0.001 | 19.603 | (13.095,26.110) | <0.001 | 18.798 | (12.300,25.296) | <0.001 |
|  | **L5** | -2.174 | (-3.149,-1.199) | <0.001 | -2.811 | (-3.741,-1.881) | <0.001 | -2.228 | (-3.120,-1.337) | <0.001 | -2.157 | (-3.047,-1.267) | <0.001 |
|  | **M10** | 0.572 | (0.342,0.802) | <0.001 | 0.169 | (-0.062,0.399) | 0.152 | -0.066 | (-0.289,0.158) | 0.563 | -0.085 | (-0.307,0.138) | 0.455 |

Model 1: adjusted for age, sex and race.

Model 2: adjusted for age, sex, race, BMI, income, education levels, sleep duration, daily energy intake, regular exercises, current smoker, and current drinker.

Model 3: adjusted for age, sex, race, BMI, daily energy intake, education levels, sleep duration, regular exercises, current smoker, current drinker, income, self-reported diabetes, self-reported hypertension, self-reported hyperlipidemia, take medication for diabetes, take medication for hypertension and take medication for cholesterol.

**Supplementary Table 6：Associations of RAR parameters with cognitive function excluding participants** **with 6 hours or less sleep duration. (N=1376)**

|  |  | **Unadjusted** | | | **Model 1** | | | **Model 2** | | | **Model 3** | | |
| --- | --- | --- | --- | --- | --- | --- | --- | --- | --- | --- | --- | --- | --- |
|  |  | β | 95%CI | P-value | β | 95%CI | P-value | β | 95%CI | P-value | β | 95%CI | P-value |
| **CERAD W-L** | **IS** | 2.555 | (-0.236,5.347) | 0.073 | 3.199 | (0.567,5.830) | 0.017 | 2.291 | (-0.325,4.907) | 0.086 | 2.227 | (-0.384,4.838) | 0.094 |
|  | **IV** | -3.916 | (-5.564,-2.269) | <0.001 | -1.384 | (-3.008,0.240) | 0.095 | -1.253 | (-2.863,0.356) | 0.127 | -1.118 | (-2.733,0.497) | 0.175 |
|  | **RA** | 6.874 | (3.491,10.256) | <0.001 | 4.803 | (1.578,8.027) | 0.004 | 2.762 | (-0.487,6.010) | 0.096 | 2.446 | (-0.823,5.715) | 0.142 |
|  | **L5** | -0.264 | (-0.801,0.274) | 0.336 | -0.474 | (-0.980,0.032) | 0.066 | -0.264 | (-0.761,0.234) | 0.299 | -0.248 | (-0.746,0.250) | 0.329 |
|  | **M10** | 0.357 | (0.264,0.449) | <0.001 | 0.169 | (0.074,0.263) | <0.001 | 0.130 | (0.032,0.228) | 0.009 | 0.121 | (0.023,0.219) | 0.016 |
| **AFT** | **IS** | 2.042 | (-0.277,4.362) | 0.084 | 2.111 | (-0.169,4.392) | 0.070 | 0.594 | (-1.648,2.835) | 0.603 | 0.482 | (-1.753,2.718) | 0.672 |
|  | **IV** | -2.625 | (-3.998,-1.251) | <0.001 | -0.871 | (-2.279,0.537) | 0.225 | -0.444 | (-1.825,0.937) | 0.529 | -0.280 | (-1.665,1.105) | 0.691 |
|  | **RA** | 8.852 | (6.089,11.615) | <0.001 | 7.179 | (4.427,9.931) | <0.001 | 4.820 | (2.067,7.574) | <0.001 | 4.204 | (1.433,6.974) | 0.003 |
|  | **L5** | -0.951 | (-1.391,-0.511) | <0.001 | -0.975 | (-1.407,-0.543) | <0.001 | -0.758 | (-1.179,-0.337) | <0.001 | -0.696 | (-1.118,-0.274) | 0.001 |
|  | **M10** | 0.227 | (0.149,0.305) | <0.001 | 0.122 | (0.040,0.204) | 0.004 | 0.046 | (-0.038,0.130) | 0.280 | 0.038 | (-0.046,0.122) | 0.380 |
| **DSST** | **IS** | 14.544 | (7.183,21.906) | <0.001 | 16.960 | (9.961,23.960) | <0.001 | 10.021 | (3.392,16.650) | 0.003 | 9.210 | (2.660,15.761) | 0.006 |
|  | **IV** | -9.001 | (-13.375,-4.626) | <0.001 | -3.113 | (-7.464,1.239) | 0.161 | -1.404 | (-5.501,2.693) | 0.502 | -0.130 | (-4.199,3.939) | 0.950 |
|  | **RA** | 43.744 | (35.117,52.371) | <0.001 | 40.861 | (32.553,49.169) | <0.001 | 30.338 | (22.294,38.382) | <0.001 | 27.826 | (19.796,35.857) | <0.001 |
|  | **L5** | -4.763 | (-6.152,-3.374) | <0.001 | -5.444 | (-6.758,-4.131) | <0.001 | -4.333 | (-5.567,-3.098) | <0.001 | -4.097 | (-5.322,-2.872) | <0.001 |
|  | **M10** | 0.873 | (0.627,1.119) | <0.001 | 0.440 | (0.188,0.693) | <0.001 | 0.096 | (-0.154,0.345) | 0.452 | 0.055 | (-0.192,0.302) | 0.662 |

Model 1: adjusted for age, sex and race.

Model 2: adjusted for age, sex, race, BMI, income, education levels, sleep duration, daily energy intake, regular exercises, current smoker, and current drinker.

Model 3: adjusted for age, sex, race, BMI, daily energy intake, education levels, sleep duration, regular exercises, current smoker, current drinker, income, self-reported diabetes, self-reported hypertension, self-reported hyperlipidemia, take medication for diabetes, take medication for hypertension and take medication for cholesterol.
